# A Review of the Fear of Hypoglycemia Literature in Children and Adolescents with Type 1 Diabetes and their Caregivers

**DOI:** 10.1007/s11892-026-01620-x

**Published:** 2026-03-23

**Authors:** Kimberly A. Driscoll, Paige J. Trojanowski, Emily R. Shaffer, Hannah Manis, Alicia Pardon, Cheyenne M. Reynolds, Sara E. Wetter-Wren, Holly K. O’Donnell, Susana R. Patton

**Affiliations:** 1 School of Medicine, Department of Pediatrics, Center for Diabetes Technology, University of Virginia, Charlottesville, VA USA; 2 Center for Health Outcomes Research and Science Delivery, Children’s National Hospital, Washington, DC USA; 3 The George Washington School of Medicine and Health Sciences, Washington, DC USA; 4 Department of Psychology, George Mason University, Fairfax, VA USA; 5 Department of Psychology, Texas Children’s Hospital, Houston, TX USA; 6 Department of Pediatrics, Division of Pediatric Psychology, Michigan Medicine, Ann Arbor, MI USA; 7 Center for Behavioral Health, Johns Hopkins All Children’s Hospital, Saint Petersburg, FL USA; 8 Department of Behavioral Medicine & Clinical Psychology, Cincinnati Children’s Hospital Medical Center, Cincinnati, OH USA; 9 School of Medicine, Department of Pediatrics, University of Colorado, Barbara Davis Center for Diabetes, Aurora, CO USA; 10 Nemours Center for Healthcare Delivery Science, Nemours Children’s Health, Jacksonville, FL USA

**Keywords:** Hypoglycemia Fear Surveys, Diabetes technology, Anxiety, Youth

## Abstract

**Purpose of Review:**

To provide an updated review of the fear of hypoglycemia (FOH) literature published from 2016 to 2025, in youth with type 1 diabetes (T1D) and their caregivers.

**Recent Findings:**

The Hypoglycemia Fear Surveys (HFS) are the most used questionnaires to assess FOH in studies conducted in the United States and internationally. The factor structure of the Parent and Child versions of the HFS have been updated to better reflect only maladaptive aspects of managing FOH. Diabetes technology studies routinely include the HFS to assess FOH as a secondary outcome. Only two randomized clinical trials (RCT) to reduce FOH have been conducted with youth with T1D and their caregivers.

**Summary:**

FOH is a common psychological complication occurring in youth with T1D and their caregivers. More research is needed to update the HFS to reflect modern T1D self-management and test interventions that aim to reduce FOH in RCTs.

## Introduction

Hypoglycemia, or blood glucose < 70 mg/dL, is the most common acute complication of type 1 diabetes (T1D). Although its incidence is decreasing especially as use of sophisticated automated insulin delivery systems (i.e., insulin pumps that deliver subcutaneous rapid acting insulin without the need for long-acting insulin) increases, hypoglycemia is considered the Achille’s heel of T1D management [1]. Prolonged or recurrent hypoglycemia is associated with cognitive dysfunction, damage to the hippocampus, neuropathy, and increased risk for dementia in the long-term [2]. Equally, or perhaps more concerning, are the immediate consequences of a severe hypoglycemic episode (i.e., a low blood glucose occurring in an individual who is unable to provide treatment independently) including loss of consciousness, seizures, coma, and death, which occurs at least once in approximately 5% of youth with T1D [3–5]. Therefore, it is not surprising that anxiety (i.e., fear of hypoglycemia; FOH) about having a hypoglycemic event occurs in some individuals with T1D and their family members. Indeed, FOH is recognized as an “underestimated” [6] problem that serves as a barrier to optimal T1D self-management; clinical care tends to focus on hyperglycemia without comprehensive assessment of its underlying causes, lack of routine FOH screening, and lack of availability of clinical cut points to assist T1D providers with determining when FOH is impairing.

FOH is a psychological condition, and in its most severe presentation is akin to a phobia of having a low blood glucose. However, like many anxiety conditions, it occurs on a continuum from maladaptive (e.g., extreme hypervigilance to glucose values, not allowing children to attend sleepovers, caregiver leaving the workforce to be immediately available if a hypoglycemic event occurs) to adaptive (i.e., similar to the Yerkes-Dodson law in which an optimal amount of anxiety about low glucose leads to monitoring that does not cause impairment). Maladaptive FOH is conceptualized as extreme engagement in behaviors by an individual with T1D or their caregiver to avoid a low blood glucose and/or when either individual engages in excessive worrying about having a low blood glucose and its consequences [7, 8]. The purpose of the current article is to provide an update to the FOH literature review we published in 2016, focusing on its assessment; associations with biological, social, and psychological sequelae; and interventions [9].

## Assessment of FOH

We previously provided a summary of the development of the Hypoglycemia Fear Surveys (HFS [9]), which are the most commonly used questionnaires to assess FOH in children and adolescents with T1D and their caregivers in the United States and internationally [10–16]. However, briefly, the HFS [7] was originally developed for use with adults with T1D, and it was then modified to create separate caregiver (Parent Hypoglycemia Fear Survey; PHFS; for caregivers of children and adolescents *≥* 8 years of age) and child (CHFS; children and adolescents *≥* 8 years of age) self-report versions [17]. For the PHFS, each item was modified to reflect the parent’s child (e.g., “Allow my child…”). Patton and colleagues developed a HFS version for caregivers of young children < 8 years of age [18, 19] and Monzon and colleagues [20] recognized that the HFS did not adequately assess FOH that occurs at night despite worry about FOH at night [21]. Thus, using gold standard psychometric methods [22], Monzon and colleagues specifically validated items for a new PHFS subscale, Nighttime Worry, related to nighttime FOH for use with caregivers of children and adolescents with T1D. Finally, Kamps and colleagues [23] developed the Children’s Hypoglycemia Index to assess children’s (ages 8–16 years) self-reported FOH in three areas, Behavior, Situations, and General, but it is rarely used in published studies.

Several research groups opted not to use validated FOH questionnaires and instead used 1 or 2-item questions to assess FOH: “Does fear of hypoglycemia, due to the loss of glycemic control related to physical activity, keep you from practicing this activity?” [24, 25]; or the degree that the individual is (a) afraid of hypoglycemia and (b) feel the fear has a negative impact on their daily life [26]. Forsander and colleagues [27] did not specify the question they used to assess FOH but included the scale: 0 = “not scared at all” to 10 = “very scared.” We do not recommend using 1- or 2-items to assess FOH (or any psychological condition) for several reasons. First, 1-item questions cannot be assessed for reliability [28]. Second, FOH is multi-faceted and 1- or 2-items cannot capture content validity or all relevant aspects of FOH [29, 30]. Third, the likelihood of random error increases [31]. Finally, there is a lack of sensitivity for detecting change across time [32]. For these reasons, studies that did not use a validated FOH questionnaire were not included in the current review.

## HFS Factor Structures and Scoring

The original PHFS and CHFS versions each comprised 25 questions on two subscales, Behavior and Worry, which were scored separately [33] and a few early studies used factor analysis to support the two factor structure (7, 34); however, different versions of the PHFS and CHFS with varying numbers of items on each of the two subscales have been used [7, 34–37]. Other than providing Cronbach’s alphas and test-retest reliability, it appears that confirmatory factor analysis has not been conducted on the PHFS and CHFS despite their increased use in research studies and poor to questionable Cronbach’s alphas for the Behavior subscale [7, 8, 33, 35].

To improve understanding of the FOH construct, Shepard and colleagues [35] conducted separate exploratory factor analysis of the original PHFS and CHFS in a research sample of children with T1D and their caregivers. It is important to note that the PHFS and CHFS used in their exploratory factor analyses each comprised 25 items (10 items on the Behavior subscale and 15 on the Worry subscale]. The exploratory factor analyses revealed four factors for both the parent and child versions, Maintain High Blood Glucose; Avoid/Prevent Low Blood Glucose; Helplessness/Worry About Low Blood Glucose; and Worry About Negative Social Consequences [35]. However, Cronbach’s alphas for the Avoid/Prevent Low Blood Glucose factor were poor [35] calling into question whether the items on this factor capture FOH behaviors [36]. Additionally, caregivers with high scores on the Avoid/Prevent Low Blood Glucose subscale had children with lower HbA1c, suggesting that some of the items represent adaptive behaviors, not fear. For example, items such as “Carry fast acting sugar,” “Reduce insulin when blood glucose could be low,” and “Eat at first symptom of a low blood glucose” are medically appropriate and recommended behaviors to prevent or treat low blood glucose. The exploratory factor analyses conducted by Shepard and colleagues also revealed problematic fit statistics with several items loading onto more than one factor. In addition, some items had item-factor correlations that were less than the minimally acceptable value of 0.40 [35].

Based on Shepard and colleagues’ exploratory factor analyses, we support their recommendation to avoid using a Total score for the 25-item version. Instead, each of the three FOH subscales, Maintain High Blood Glucose; Helplessness/Worry About Low Blood Glucose; and Worry About Negative Social Consequences, should be calculated and interpreted separately [35]. However, the factor structure and psychometrics of the Total score without the Avoid/Prevent Low Blood Glucose items should be examined in future research. Moreover, the Avoid/Prevent Low Blood Glucose subscales, while informative for adaptive behaviors, should be used with caution given their low internal consistencies [35]. Indeed, Shepard and colleagues suggested, and we agree, that the items on the Avoid/Prevent Low Blood Glucose subscale should be individually reviewed with children with T1D and/or their caregivers to determine if any of the individual items are problematic or if they are adaptive. For example, “Reduce insulin when blood glucose could be low” is an item that could be adaptive if the low is confirmed by a blood glucose meter or continuous glucose monitor reading. However, it could be maladaptive if the child’s or caregiver’s definition of a low differs from the conventional definition of a low. For example, it is not uncommon for individuals with T1D to develop a “safe” glucose number, which is higher than the medically recommended target range of 70–180 mg/dL. Rather, this “safe” number is an individual’s preferred minimum glucose number for administering insulin [38, 39].

## Clinical Utility of the PHFS and CHFS

In a follow-up study to Shepard and colleagues’ exploratory factor analyses of the PHFS and CHFS in a research sample, O’Donnell and colleagues conducted confirmatory factor analyses on the PHFS and CHFS, which further demonstrated the poor reliability and internal consistency of the Avoid/Prevent Low Blood Glucose subscale in a large clinical sample of children and caregivers [36]. Thus, O’Donnell and colleagues [36] recommended that the Avoid/Prevent Low Blood Glucose subscale should be used only to determine if children and caregivers engage in adaptive behaviors related to FOH. Although the 3 subscales based on exploratory and confirmatory factor analyses of the PHFS and CHFS are an advancement to the FOH science [35, 36], the 2 subscale versions (i.e., Behavior and Worry) continue to be used in research studies [15–42]. The clinical utility of the PHFS, CHFS, and HFS-YC is limited by a general lack of clinical cut-points to determine whether a given score warrants additional assessment to determine functional impairment and whether intervention is needed. However, O’Donnell and colleagues [37] are the first to propose a preliminary cut-point of *≥* 7 for the 3-item CHFS Maintain High Blood Glucose subscale; a PHFS cut-point could not be confirmed. More research is needed to establish cut-points for the CHFS Worry/Helplessness About Low Blood Glucoses and Negative Social Consequences of a Low Blood Glucose subscales and all PHFS and HFS-YC subscales.

## Associations Between FOH and Child Sociodemographic and T1D Clinical Characteristics

Several studies examined associations of caregiver FOH with children’s T1D clinical characteristics; however, there is little overlap across studies in the T1D clinical variables collected. One of the most important findings is the continued [9] lack of a significant association between HbA1c and caregivers’ FOH or children’s FOH [43] with one exception. Pate and colleagues found that as children’s HbA1c increased, both mothers’ and fathers’ PHFS Worry subscale scores significantly increased [14]. The associations between FOH and T1D self-management behaviors or insulin regimen used (multiple daily injections v. insulin pump) are not examined as frequently as is FOH with HbA1c. However, Aalders and colleagues found that as caregiver FOH increases, so does self-reported frequency of blood glucose checking [13]. In contrast, Muradoğlu and colleagues did not find a significant association between caregiver-reported FOH and the frequency of blood glucose checking [44]. Additionally, caregiver FOH does not appear to be associated with their children’s insulin regimen [10, 13, 45]. Similarly, the associations of FOH with other T1D clinical and sociodemographic variables are mixed. Children’s T1D duration and caregiver FOH are generally not related [13, 14, 44–46] nor are children’s age and caregiver or children’s FOH [13, 43, 46]. However, at least one study found that FOH increases for parents of younger children [14], and Muradoğlu and colleagues found that FOH increases for fathers of young children but not mothers [44]. Taken together, more studies are needed to examine the associations of sociodemographic and T1D clinical variables with caregiver and children’s experiences of FOH.

## Associations Between FOH and Psychological Sequelae Related to T1D

Several studies have examined a variety of psychological sequelae associated with T1D in relation to FOH including anxiety and depressive symptoms, stigma, self-efficacy, and caregiver stress. Pate and colleagues found that as state and trait anxiety increased, so did worry about FOH in mothers and fathers [14]. Caregiver FOH increases as trait anxiety and generalized anxiety symptoms increase [44], and child FOH increases as depressive symptoms increase [43]. In addition, higher FOH is associated with higher parenting stress in both mothers and fathers, but not their self-efficacy [12]. Finally, as FOH increases, so do perceptions of T1D-related stigma in adolescents with T1D [47]. Similar to the lack of research examining FOH in the context of sociodemographic and T1D clinical variables, more research is needed to characterize whether FOH occurs comorbidly with psychological conditions including those on the anxiety and depression continuums.

## Associations Between FOH and Sleep

Higher caregiver FOH is associated with poorer caregiver [10, 48] and child sleep quality and more frequent caregiver-initiated nighttime blood glucose checks [10, 48] using self- or caregiver-proxy reported questionnaires; however, when using objectively measured sleep data via actigraphy, a significant association between caregiver FOH Worry and child sleep duration, latency, or efficiency has not been established [49]. Abitbol and colleagues reported that nearly 10% of caregivers who experienced FOH performed ≥ 2 blood glucose checks per night; 18% of caregivers who experienced FOH reported that they shared a room with their child, and ≥ 1 time per week, 26% of caregivers reported that they experienced “very bad” or “fairly bad” sleep quality because of FOH. Eighteen percent of caregivers also reported that they had difficulty staying awake during the day ≥ 1 time per week. Higher adolescent self-reported FOH (CHFS Worry scores) is associated with reduced self-reported sleep duration and more sleep disturbances [50]. FOH (CHFS Total scores) is associated with shorter sleep duration in adolescents who do not use a CGM and in those who use it < 50% of the time. Taken together, adolescents with T1D and their caregivers are at-risk for general and FOH-related sleep disturbances [51], which if reduced has the potential to improve sleep quality [48].

## Associations Between FOH and CGM Use

Only one randomized controlled clinical trial (RCT) has been published examining whether using CGMs reduce FOH as the primary aim of the study [52]. Burckhardt and colleagues [52] conducted a 3-month long crossover study in which children ages 2–12 years were randomized to use either the Dexcom G5^®^ and remote monitoring or multiple daily injections (MDI); caregiver FOH was the primary outcome. Caregivers whose children wore CGMs experienced significantly greater reductions in PHFS Total, Behavior, and Worry scores after three months compared to caregivers of children who used MDI.

Most studies examining changes in PHFS and CHFS scores in the context of using CGMs show statistically significant decreases in scores within short-term longitudinal [11, 53]; longitudinal, but within subjects analyses only [15]; or cross-sectional [54] designs. Notably, Al Hayek and colleagues reported that adolescent CGM users’ CHFS Behavior scores decreased across 3-months, but the data they reported show that CHFS Behavior scores increased. Importantly, these results should be interpreted with caution given that some of the items on the Behavior subscale reflect adaptive behaviors. It is not known if these findings would hold if only the items reflecting intentional maintenance of high blood glucose were used. Studies describing changes in FOH for caregivers and youth with established T1D are described in Table 1.

**Table 1:** Studies examining FOH in the context of using CGM

Authors and Country	*N*	Child age	Study Design	CGM	Baseline	Summary of Change
Al Hayek et al. (2017) [11] Saudi Arabia	*N*=47	13–19 yrs	3-mo longitudinal; within subjects and insulin pump users v. MDI	Abbott Freestyle Libre	All participants: CHFS M Behavior (item) = 1.91 ± 0.39 M Worry (item) = 1.95 ± 0.38 Insulin Pump Users: M Behavior (item) = 1.8 ± 0.33 M Worry (item) = 1.88 ± 0.344 MDI: M Behavior (item) = 1.97 ± 0.46 M Worry (item) = 2.02 ± 0.42	All participants: CHFS M Behavior (item) = 2.1 ± 0.44* M Worry (item) = 1.81 ± 0.31* Insulin Pump Users: M Behavior (item) = 2.09 ± 0.4* M Worry (item) = 1.75 ± 0.27* MDI: M Behavior (item) = 2.1 ± 0.49* M Worry (item) = 1.8 ± 0.35*
Burckhardt et al. (2018) [52] Australia	Caregiver *N* = 49	M = 9.5 ± 1.9; 2–12 yrs	3-mo longitudinal; randomized, crossover	Dexcom^®^ G5 + remote monitoring	PHFS M Behavior = 24.3 ± 5.0 M Worry = 30.6 ± 12.4 M Total = 54.9 ± 14.7	Dexcom^®^ G5 M Behavior = 20.6 (19.1–22.1)*** M Worry = 24.1 (20.8–27.3)*** M Total = 44.7 (40.5–48.9)*** MDI M Behavior = 23.9 (22.4–25.4) M Worry = 29.3 (26.0–32.5.0.5) M Total = 53.2 (49.0–57.4.0.4)
Burckhardt et al. (2019) [53] Australia	Caregiver *n* = 60 Youth *n* = 38	M = 14.4 ± 3.0 yrs	2-mo longitudinal; within subjects	Dexcom^®^ G5 Medtronic^®^ G2 and MiniLink	CHFS M Behavior = 18.5 ± 6.0 M Worry = 18.9 ± 14.1 M Total = 37.4 ± 17.6 PHFS M Behavior = 21.8 ± 6.4 M Worry = 28.2 ± 11.2 M Total = 50.2 ± 15.0	CHFS M Behavior = 18.6 ± 5.6 M Worry = 14.5 ± 10.4* M Total = 33.1 ± 14.2 PHFS M Behavior = 44.3 ± 15.3 M Worry = 24.2 ± 12.2** M Total = 44.3 ± 15.3**
Glocker et al. (2022) [54] Switzerland	Caregiver *n* = 49 Youth *n* = 59	M = 13.8 ± 3.2 yrs	Cross-sectional	Not specifically reported but rtCGM and isCGM	PHFS rtCGM Mdn Total = 45 (34.0, 54.0) PHFS isCGM Mdn Total = 39 (26.0, 49.0) CHFS rtCGM Mdn Total = 32 (26.5, 39.0) CHFS isCGM Mdn Total = 32 (23.0, 40.0) CHFS M Behavior = 18.51 ± 5.7 M Worry = 13.73 ± 8.9 M Total = 32.2 ± 11.9 PHFS M Behavior = 20.63 ± 6.5 M Worry = 17.29 ± 9.9 M Total = 37.9 ± 14.6	Caregiver HFS scores significantly greater than children’s scores on Total (*p* = 0.047) and Worry (*p* = 0.011) No significant differences in caregiver or child scores when comparing those using rtCGM to isCGM
Ng et al. (2019) (15) United Kingdom	Caregivers *n* = 16 Youth *n* = 11	Med = 13.5 yrs; 2–17 yrs	12-mo longitudinal; within subjects	Dexcom^®^ G4	PHFS M Behavior = 38.38 M Worry = 60.31 M Total = 98.69 CHFS M Behavior = 40.88 M Worry = 56.00 M Total = 97.38	PHFS Follow-up scores M Behavior = 25.00*** M Worry = 41.69*** M Total = 66.69*** CHFS Follow-up scores M Behavior = 24.13** M Worry = 35.61** M Total = 59.75**

*p<0.05; **p < 0.01; ***p < 0.001; M = Mean; Mdn = Median

Youngkin and colleagues [41] conducted a 3-year observational study of psychological and social factors that contribute to glycemia, and they examined FOH change in caregivers of young children ages 5–9 years with new-onset T1D whose children initiated CGM use during the study. They examined their data across two time points: CGM initiation during the 6 months between Baseline and 6-months (T1-T2) and 6-months and 12-months (T2-T3). After controlling for T1D duration, only caregivers’ PHFS Behavior scores significantly decreased if their children had started CGM in the 6-months between T1 and T2 compared to children who did not start CGM during the same 6-months [41]. The data from this study should be reexamined to determine whether PHFS Behavior decreases if only the items that reflect intentional maintenance of high blood glucose are used, which would further strengthen their conclusion that initiation of CGM at T1D onset mitigates FOH.

Cross-sectional studies of FOH reveal mixed findings related to CGM use and FOH. Abitbol and colleagues found that CGM use was associated with increased FOH among caregivers of children with T1D [10]. In contrast, Van Name and colleagues [21] did not find any difference in FOH between CGM users and non-users in a very large sample of caregivers from the T1D Exchange (*N* = 549 children < 7 years of age). Similarly, Muradoğlu and colleagues found no association between FOH and CGM use in caregivers of children ages 2–17 years with T1D [44].

Taken together, it appears that youth with T1D and their caregivers generally experience decreases in FOH when CGMs are used. However, whether long-term use of CGM assists in the maintenance of reduced FOH across time has yet to be determined. Although Ng and colleagues’ 12-month long study suggests this may be the case [15], more studies are needed to determine the impact of CGM use on FOH. Existing studies limit broad conclusions about CGM and FOH because of small sample sizes [11, 15, 53, 54] or within subjects [15, 53], cross-sectional [21, 54] or short-term longitudinal [11, 53] designs. CGM studies examining FOH are described in detail in Table 1.

## Associations Between FOH and Use of Insulin Pump Technology

There have been marked improvements in insulin pump and CGM technologies in the last decade, including CGM data sharing and automated hybrid closed-loop (HCL) pumps that allow CGM-pump communication to adjust basal insulin delivery based on rising or falling glucose.

All HCL systems studies included in the current review examined changes in FOH as a *secondary* outcome. Of these studies, three used the HFS-II [55–57], which was validated for use in adults with T1D, not youth with T1D or their caregivers, and one study used the Children’s Hypoglycemia Index [58], which is very rarely used in diabetes technology and patient-reported outcomes studies. None of the studies using the HFS-II or Children’s Hypoglycemia Index found statistically significant changes across 6-months of using an insulin pump system.

Only one insulin pump technology study of very young children has been published using the HFS-YC [59], a version of the HFS specifically validated for use with caregivers of young children with T1D. Statistically significant changes in FOH Total scores occurred in caregivers whose children were randomized to HCL, compared to those randomized to the sensor augmented pump group.

Despite recommendations that only the PHFS and CHFS Maintain High Blood Glucose, Helplessness/Worry About Low Blood Glucoses, and Negative Social Consequences of Low Blood Glucose subscales be used, most recent HCL studies continue to report scores based on its original conceptualization (two subscales: Behavior and Worry, and Total score). Overall, longitudinal HCL studies lasting 3–12 months that reported Behavior and Worry subscale scores showed statistically significant changes in FOH with both caregiver and youth FOH decreasing across time [16, 60–64]. In contrast, Berget and colleagues [64] and Cobry and colleagues [65, 66] are the only investigators to provide data for the PHFS and CHFS Maintain High Blood Glucose, Helplessness/Worry About Low Blood Glucoses, and Negative Social Consequences of Low Blood Glucose subscales in HCL studies [65, 66]. In a 4-month long study, Cobry and colleagues found no statistically significant changes in FOH between youth ages 6–13 years of age or their caregivers randomized to either closed-loop control or sensor augment pump [65]. However, in a 6-month long prospective, observational, within subjects study, Cobry and colleagues found that only scores on the Helplessness/Worry About Low Blood Glucoses and Negative Social Consequences of Low Blood Glucose subscales significantly decreased at 3-months for youth and their caregivers, whereas at 6-months, scores on Maintain High Blood Glucose, Helplessness/Worry About Low Blood Glucoses, and Negative Social Consequences of Low Blood Glucose significantly decreased for both youth with T1D and their caregivers [66]. Berget and colleagues found that both caregiver and youth FOH decreased across 12 months in their prospective, observational study [64].

It is important to note that none of the technology studies were designed to assess FOH change as a primary endpoint and as a result, they may not have been powered sufficiently to detect changes. Overall, results of studies describing FOH change in the context of using T1D insulin pump technology are mixed for caregivers and youth, with some studies finding significant decreases in FOH [57, 59–63, 66] and others finding no statistically significant changes in FOH [16, 40, 41, 55, 56, 58, 63, 65, 67–69]. Similar to CGM studies, studies examining FOH in the context of using an insulin pump system are limited regarding generalizability because of inappropriate use of the HFS-II [55–57], small sample sizes [16, 56, 58, 61, 63, 66–69], or within subjects [16, 58, 59, 61–63, 66] or short-term longitudinal designs. Insulin pump technology studies examining FOH are described in detail in Table 2.

**Table 2:** Studies examining FOH in the context of using insulin pump technology

Authors and Country	*N*	Child age	Study Design	Insulin Pump	Baseline	Summary of Change
Abraham et al. (2025) [55] Australia	Youth + Young Adults *N* = 46	M = 16.2 ± 2.5; 12–25 yrs	6-mo longitudinal RCT	CSII + CGM v. Advanced hybrid closed loop (AHCL)	HFS-II (mean item) CSII Total = 1.0 ± 0.7 HFS-II (mean item) AHCL Total = 1.3 ± 1.0	HFS-II (mean item) CSII Total = 0.8 ± 0.7 HFS-II (mean item) AHCL Total = 0.9 ± 0.9
Berget et al. (2025) [64] United States	Youth + Young Adults *N* = 141	M = 12 ± 6; 2–21 yrs	12-mo longitudinal; observational	Omnipod 5	CHFS LSM Maintain High = 4.8 (0.3) LSM Worry/Helplessness = 10.8 (0.7) LSM Social Consequences = 6.0 (0.5) PHFS LSM Maintain High = 5.0 (0.2) LSM Worry/Helplessness = 18.2 (0.7) LSM Social Consequences = 3.3 (0.3)	CHFS – 3 mos LSM Maintain High = 4.6 (1.1) LSM Worry/Helplessness = 9.6 (0.7) LSM Social Consequences = 6.0 (0.5) PHFS – 3 mos LSM Maintain High = 4.4 (0.2) LSM Worry/Helplessness = 15 (0.7) LSM Social Consequences = 2.2 (0.3) CHFS – 6 mos LSM Maintain High = 4.2 (0.3) LSM Worry/Helplessness = 9.4 (0.7) LSM Social Consequences = 5.8 (0.5) PHFS – 6 mos LSM Maintain High = 3.9 (0.2) LSM Worry/Helplessness = 15.6 (0.7) LSM Social Consequences = 2.4. (0.3) CHFS – 9 mos LSM Maintain High = 3.9 (0.3) LSM Worry/Helplessness = 8.8 (0.7) LSM Social Consequences = 6.0 (0.5) PHFS – 9 mos LSM Maintain High = 4.1 (0.2) LSM Worry/Helplessness = 14.4 (0.7) LSM Social Consequences = 2.5 (0.3) CHFS − 12 mos LSM Maintain High = 3.7 (0.3) LSM Worry/Helplessness = 8.2 (0.7) LSM Social Consequences = 6.0 (0.5) PHFS − 12 mos LSM Maintain High = 3.8 (0.2) LSM Worry/Helplessness = 14.9 (0.7) LSM Social Consequences = 2.6 (0.3)
Bisio et al. (2021) [67] United States	Child-caregiver dyads *N* = 13	M = 9.1 ± 0.9; 7–10 yrs	8 weeks	Control IQ, Tandem t: slim X2 v. personal pump + Dexcom^®^ G6	CHFS M Behavior = 21.25 ± 4.33 M Worry = 16.33 ± 5.63 M Total = 37.58 ± 5.50 PHFS M Behavior = 32.45 ± 4.93 M Worry = 32.00 ± 12.35 M Total = 64.45 ± 17.12	CHFS – CLC M Behavior = 21.67 ± 5.45 M Worry = 14.75 ± 6.18 M Total = 36.42 ± 9.08 CHFS – SAP M Behavior = 23.17 ± 5.18 M Worry = 15 ± 5.44 M Total = 38.17 ± 8.83 PHFS – CLC M Behavior = 22.09 ± 7.83 M Worry = 20.64 ± 10.89 M Total = 42.73 ± 16.54 PHFS – SAP M Behavior = 33.09 ± 5.45 M Worry = 30.45 ± 14.51 M Total = 63.55 ± 18.3
Cobry et al. (2021) [65] United States	Caregiver-youth dyads *N* = 101	M = 11.2 ± 2.1; 6–13 yrs	16-weeks; longitudinal RCT	Control IQ, Tandem t: slim X2 v. personal pump + Dexcom^®^ G6	CHFS - CLC M Behavior = 49 ± 13 M Maintain High = 32 ± 22 M Worry = 25 ± 20 M Worry/Helplessness = 24 ± 18 M Social Consequences = 25 ± 23 M Total = 35 ± 14 CHFS - SAP M Behavior = 51 ± 15 M Maintain High = 37 ± 21 M Worry = 24 ± 18 M Worry/Helplessness = 23 ± 16 M Social Consequences = 27 ± 25 M Total = 35 ± 14 PHFS - CLC M Behavior = 58 ± 14 M Maintain High = 36 ± 18 M Worry = 34 ± 18 M Worry/Helplessness = 42 ± 19 M Social Consequences = 19 ± 20 M Total = 44 ± 14 PHFS - SAP M Behavior = 61 ± 15 M Maintain High = 43 ± 16 M Worry = 40 ± 22 M Worry/Helplessness = 49 ± 25 M Social Consequences = 22 ± 22 M Total = 48 ± 17	CHFS – CLC – 16 weeks M Behavior = 47 ± 11 M Maintain High = 29 ± 20 M Worry = 21 ± 15 M Worry/Helplessness = 21 ± 15 M Social Consequences = 20 ± 17 M Total = 31 ± 12 CHFS – SAP – 16 weeks M Behavior = 50 ± 14 M Maintain High = 34 ± 19 M Worry = 26 ± 17 M Worry/Helplessness = 26 ± 16 M Social Consequences = 26 ± 20 M Total = 36 ± 14 PHFS – CLC – 16 weeks M Behavior = 47 ± 15 M Maintain High = 28 ± 17 M Worry = 29 ± 15 M Worry/Helplessness = 37 ± 17 M Social Consequences = 14 ± 15 M Total = 44 ± 14 PHFS – SAP – 16 weeks M Behavior = 56 ± 15 M Maintain High = 40 ± 15 M Worry = 37 ± 16 M Worry/Helplessness = 44 ± 19 M Social Consequences = 21 ± 14 M Total = 45 ± 13
Cobry et al. (2022) [60] United States	Caregiver-youth dyads *N* = 101 Youth with clinically elevated Pittsburgh Sleep Quality Index scores	M = 11.2 ± 2.1; 6–13 yrs	16-weeks; longitudinal RCT	Control IQ, Tandem t: slim X2 v. personal pump + Dexcom^®^ G6	CHFS Mdn Behavior = 30 (26–33) Mdn Worry = 27 (23–34) Mdn Total = 57 (51–67) PHFS Mdn Behavior = 33 (29–37) Mdn Worry = 37 (34–47) Mdn Total = 73 (63–81)	CHFS Mdn Behavior = 30 (24–32) Mdn Worry = 27 (21–33) Mdn Total = 55 (48–67)*** PHFS Mdn Behavior = 29 (24–32)*** Mdn Worry = 33 (29–40)** Mdn Total = 65 (54–74)***
Cobry et al. (2024) [66] United States	Caregiver-youth dyads *N* = 39	M = 11.1 ± 3.6; 6–13 yrs	6-mo longitudinal within subjects	Control IQ, Tandem t: slim X2 v. personal pump + Dexcom^®^ G6	CHFS LSM Behavior = 18.9 (1.0) LSM Maintain High = 3.8 (0.5) LSM Worry = 15.4 (1.3) LSM Worry/Helplessness = 10.4 (0.9) LSM Social Consequences = 3.5 (0.4) LSM Total = 34.4 (2.0) PHFS LSM Behavior = 22.5 (1.2) LSM Maintain High = 4.2 (0.5) LSM Worry = 20.8 (1.3) LSM Worry/Helplessness = 17.6 (1.1) LSM Social Consequences = 3.2 (0.4) LSM Total = 43.4 (2.2)	CHFS – 3-mo LSM Behavior = 20.0 (1.0) LSM Maintain High = 4.1 (0.5) LSM Worry = 12.1 (1.3)* LSM Worry/Helplessness = 8.3 (0.9)* LSM Social Consequences = 2.3** (0.4) LSM Total = 32.1 (1.9) CHFS – 6-mo LSM Behavior = 17.5 (1.0)* LSM Maintain High = 3.4 (0.5) LSM Worry = 11.5 (1.3)** LSM Worry/Helplessness = 8.0 (0.9)** LSM Social Consequences = 2.2** (0.4) LSM Total = 29.0 (2.0) PHFS – 3-mo LSM Behavior = 20.6 (1.2) LSM Maintain High = 4.1 (0.5) LSM Worry = 15.9 (1.3)** LSM Worry/Helplessness = 13.2 (1.1)** LSM Social Consequences = 2.7 (0.4) LSM Total = 36.4 (2.2)** PHFS – 6-mo LSM Behavior = 19.5 (1.3)** LSM Maintain High = 3.5 (0.5) LSM Worry = 15.2 (1.5)** LSM Worr/Helplessness = 13.3 (1.2)** LSM Social Consequences = 1.9 (0.5) LSM Total = 34.7 (2.4)**
de Beaufort et al. (2022) [59] Multiple European countries	Youth *N* = 74	M = 5 ± 2; 1–7 yrs	RCT crossover	Dana Diabecare RS + Dexcom^®^ G5 with and without CamAPS FX algorithm	HFS-YC M Total = 72.4 ± 14.9 M Behavior = 35.4 ± 5.9 M Worry = 36.9 ± 11.2	HCL HFS-YC M Total = 64.6 ± 2.6*** M Behavior = 33.4 ± 5.8** M Worry = 31.2 ± 8.7*** SAP M Total = 68.9 ± 12.7 M Behavior = 34.9 ± 6.6 M Worry = 34.2 ± 9.2
De Meulemeester, et al. (2025) [62] Belgium	Youth *N* = 114	M = 12.0 ± 3.2; 6–17 years	12-mo longitudinal; observational; within subjects	Control IQ, Tandem t: slim X2	CHFS LSM Behavior = 17.8; 16.1–19.50 LSM Worry = 16.1; 13.6–18.6 PHFS LSM Behavior = 24.3; 21.4–27.3 LSM Worry = 25.0; 21.6–28.4	CHFS LSM Behavior = 18.1; 16.2–20.0 LSM Worry = 15.0; 12.5–17.5 PHFS LSM Behavior = 19.8; 16.8–22.8*** LSM Worry = 20.3; 17.0–23.5.0.5***
DuBose et al.^++^ (2021) [68] United States	Caregivers *N* = 20	Not reported	12-mo longitudinal within subjects	Medtronic MiniMed™ 670G + GuardianLink	HFS^+^ Mdn Total = 45 (35, 58) Mdn Behavior = 58 (45, 70) Mdn Worry = 35 (26, 53)	6 weeks Mdn Total = 41 (27, 50) Mdn Behavior = 48 (40, 61) Mdn Worry = 32 (19, 44) 6 mo Mdn Total = 40 (30, 48) Mdn Behavior = 45 (32, 59) Mdn Worry = 35 (15, 43) 12 mo Mdn Total = 38 (28, 46) Mdn Behavior = 45 (36, 57) Mdn Worry = 30 (22, 43)
Hood et al. (2024) [57] United States	Caregivers *N* = 96	M = 4.17 ± 1.23; 2–5 yrs	26-weeks longitudinal RCT	Control IQ, Tandem t: slim X2 v. standard care	Standard Care → Control IQ HFS-II M Total = 41.9 ± 16.4 Control IQ → Control IQ HFS-II M Total = 44.0 ± 17.5	Standard Care → Standard Care HFS-II − 13-weeks M Total = 38.7 ± 15.3 Standard Care → Control IQ HFS-II − 26-weeks M Total = 35.6 ± 12.8 Control IQ → Control IQ HFS-II − 13-weeks M Total = 37.5 ± 17.0 HFS-II − 26-weeks M Total = 34.8 ± 16.9 *p* < 0.05
Jalilova et al. (2024) [58] Turkey	Children + Adolescents *N* = 41	M = 12.5 ± 3.2; 6–18 yrs	6-mo longitudinal within subjects	Medtronic MiniMed™ 780G	Mdn Specific situations = 11 Mdn General fears = 18 Mdn Behavior = 10 Mdn Total = 40	CHI – 6-mo Mdn Specific situations = 10 Mdn General fears = 18 Mdn Behavior = 10 Mdn Total = 38
Kessler et al. (2025) [63] France	Not reported	Not reported	12-mo longitudinal; observational; within subjects	Medtronic MiniMed™ 780G	CHFS M Total = 31.1 PHFS M Total = 35.9	CHFS – 6 mo M Total = 29.2 CHFS – 12 mo M Total = 27.1 PHFS – 6 mo M Total = 28.9** PHFS – 12 mo M Total = 28.2**
Kudva et al. (2021) [56] United States	Adolescents + Young Adults *N* = 31	14–24 yrs	26-weeks longitudinal; RCT	Control IQ, Tandem t: slim X2 v. personal pump + Dexcom^®^ G6	HFS-II CLC M Behavior = 56 ± 15 M Maintain High = 36 ± 23 M Worry = 23 ± 16 M Total = 36 ± 12 SAP M Behavior = 50 ± 16 M Maintain High = 29 ± 21 M Worry = 30 ± 16 M Total = 38 ± 13	CLC – 13 weeks M Behavior = 52 ± 13 M Maintain High = 34 ± 20 M Worry = 20 ± 16 M Total = 33 ± 11 SAP – 26 weeks M Behavior = 50 ± 15 M Maintain High = 28 ± 20 M Worry = 25 ± 17 M Total = 35 ± 13 CLC – 13 weeks M Behavior = 53 ± 14 M Maintain High = 33 ± 21 M Worry = 19 ± 14 M Total = 32 ± 10 SAP – 26 weeks M Behavior = 45 ± 14 M Maintain High = 26 ± 22 M Worry = 22 ± 18 M Total = 31 ± 16
Ng et al. (2022) [16] United Kingdom	Caregivers *n* = not reported Youth *n* = 39	M = 11.8 ± 4.4; 2–18 yrs	3-mo longitudinal; observational; within subjects	Control IQ, Tandem t: slim X2 or CamAPS FX	PHFS M Behavior = 31.9 ± 8.5 M Worry = 31.7 ± 13.2 M Total = 63.8 ± 19.6 CHFS M Behavior = 34.0 ± 6.6 M Worry = 40.2 ± 11.1 M Total = 73.1 ± 17.2	PHFS – 3-mo M Behavior = 18.9 ± 8.3*** M Worry = 20.3 ± 10.8** M Total = 40.2 ± 14.2** CHFS – 3-mo M Behavior = 27.5 ± 6.7* M Worry = 31.6 ± 12.0* M Total = 56.2 ± 14.4**
Schierloh et al. (2024) [69] Luxembourg	Caregivers *n* = 30 Youth *n* = 32	M = 10.5 ± 2.3; 6–14 yrs	5-weeks longitudinal; RCT, crossover	Medtronic MiniMed 640G + SmartGuard *or* other pump, + Abbott Freestyle Libre 1^®^ isCGM	PHFS adjusted M = 40.67 Children’s Hypoglycemia Index adjusted mean = 55.1 Raw scores not reported	Raw scores not reported; No statistically significant difference between groups.
Verbeeten et al. (2021) [40] Canada	Caregivers *n* = 122 Youth *n* = 95	5–18 yrs	12-mo longitudinal RCT	Enlite™ + Paradigm™ Veo™ Insulin Pump Medtronic Canada	CHFS M Behavior = 21.1 ± 5.9 M Worry = 17.9 ± 11.9 PHFS M Behavior = 20.7 ± 7.5 M Worry = 23.1 ± 13.2	CHFS M Behavior = 17.2 ± 6.1*** M Worry = 11.9 ± 11.4*** PHFS M Behavior = 17.4 ± 7.4*** M Worry = 17.6 ± 10.4***
Zuijdwijk et al. (2023) [61] Canada	Caregivers *n* = 43 Youth *n* = 49	Mdn = 13.8 (11.1, 15.7); 6–18 yrs	16-week longitudinal; observational	Control IQ, Tandem t: slim X2	CHFS M Behavior = 20.4 ± 6.5 M Worry = 19.9 ± 13.1 M Total = 40.3 ± 15.4 PHFS M Behavior = 23.9 ± 5.2 M Worry = 27.4 ± 13.0 M Total = 51.3 ± 16.2	CHFS – 16 weeks M Behavior = 20.5 ± 5.3 M Worry = 15.7 ± 10.6** M Total = 36.2 ± 13.0** PHFS – 16 weeks M Behavior = 20.5 ± 6.3*** M Worry = 22.4 ± 11.1** M Total = 42.9 ± 15.4***

*p<0.05; **p < 0.01; ***p < 0.001; +unclear if caregiver version was administered; ++Youth HFS combined with adult HFS data; M = mean; Med = median; SAP = sensor augmented pump; CLC = closed loop control

Jalilova et al. (2024) [58] study reported in their text that they used the CHFS, but they actually used the Children's Hypoglycemia Index (CHI) and its subscales

## Associations Between FOH and Physical Activity

Two large studies using the CHFS found that higher adolescent-reported FOH, specifically the Behavior subscale, was associated with *more* self-reported vigorous physical activity [70, 71], whereas parent-reported FOH was not related to youth physical activity [71]. Additionally, physical activity for the purposes of T1D self-management, as measured by the Diabetes Self-Management Questionnaire (DSMQ; Arabic version), was not correlated with adolescent CHFS scores [72]. These results suggest that while youth may report FOH as a barrier to physical activity, their fear may not reliably translate to decreased physical activity. Alternatively, the physical activity subscale of the DSMQ with only 3-items may not reliably assess physical activity. An additional limitation of the DSMQ - Arabic version is that it has only been validated in type 2 diabetes. In contrast to adolescent findings, one small study of 25 preschoolers found that fewer minutes of daily moderate to vigorous physical activity and more minutes of sedentary behavior, as measured by accelerometry, were each associated with higher parent HFS Worry scores on the HFS-YC [73].

Taken together, FOH may impact physical activity differently based on child or adolescent age and self- versus parent-report, particularly when children are young and parents have more control over their activity choices. However, more research in young children with larger samples is needed. Additionally, interpretations of findings in the extant literature on FOH and exercise is compromised by use of unvalidated, single-item assessments of FOH and the cross-sectional nature of studies, thus preventing causal inferences about FOH and youth physical activity patterns from being drawn.

## Interventions to Reduce FOH

Despites decades of research establishing FOH as a psychological complication of T1D, very few interventions have been tested to reduce its occurrence in youth with T1D and/or their caregivers. Vallis and colleagues published guidance on how to treat FOH based on cognitive behavioral therapy and exposure and response prevention [39], which are the gold standard treatments for anxiety disorders including phobias [74]. O’Donnell and colleagues published a case study of successful intervention for a young adult with T1D using cognitive behavioral therapy and exposure and response prevention [38], but no such case study has been published in youth with T1D and/or their caregivers.

Patton and colleagues conducted a pilot RCT, Reducing Emotional Distress for Childhood Hypoglycemia in Parents (*REDCHiP*), demonstrating significant reductions in FOH in caregivers of young children 1 to 6 years of age who received intervention compared to a waitlist control group [75]. The *REDCHiP* intervention is based on principles of cognitive behavioral therapy including exposure and response prevention to improve distress and improve coping [75]. Patton and colleagues recently completed a larger 9-month long RCT to reduce FOH in caregivers of young children who were randomized to either *REDCHiP* or an attention control group; main outcomes from the RCT are forthcoming [76]. Importantly, caregivers acknowledged the significance of identifying and modifying cognitive distortions related to hypoglycemia and engaging in exposures when treating FOH with one caregiver commenting, “*[Exposures] helped me be better at not sitting and staring at his Dexcom all day* [77].”

O’Donnell and colleagues also conducted a small 9-month long pilot RCT, *Bring BG Down!*, to provide intervention to mothers with elevated FOH of adolescents with T1D. Participants were randomized to either the intervention group [i.e., cognitive behavioral therapy, exposure and response prevention] or no-treatment control [i.e., completing the PHFS and CHFS only] [78]. Results from *Bring BG Down!* are also forthcoming.

Finally, Güneş Kaya and colleagues completed a within subjects education intervention with children and adolescents ages 8–18 years of age to improve hypoglycemia management, awareness, and fear and HbA1c [79]. They found that Turkish youth reported statistically significant reductions in FOH as measured by a modified 32-item version of the HFS.

## Conclusion

The HFS continue to be the most used questionnaires to assess FOH in youth with T1D and their caregivers. Updates to the factor structure of the PHFS and CHFS reflect three subscales that should be individually totaled and used separately, Maintain High Blood Glucose, Worry/Helplessness About Low Blood Glucoses, and Worry About Social Consequences of Low Blood Glucose. Only one study has identified a cut-point for the CHFS and only for the Maintain High Blood Glucose subscale. The PHFS and CHFS are routinely used to assess FOH as a secondary outcome in diabetes technology studies, with most studies showing mixed results, although it appears that CGM use reduces FOH in the short-term. However, very few studies are longitudinal. Similarly, only two RCTs have been conducted with youth and their caregivers to test interventions to reduce FOH. Taken together, we offer the following recommendations for future areas of study related to FOH:

1. Revise the HFS versions to reflect modern diabetes technology. For example, specific questions could include the frequency of which individuals check CGM readings, whether pump users worry about too much insulin being delivered, and whether they have determined their own “safe” glucose number outside of the recommended range and if boluses are declined or omitted in that range.
2. Increase use of the HFS subscales, Maintain High Blood Glucose, Worry/Helplessness About Low Blood Glucoses, and Worry About Social Consequences of Low Blood Glucose, in studies to reflect the updated factor structures. When the 2 factor structure is used, investigators should interpret the Behavior subscale with caution.
3. Standardize how scores are reported [means v. medians v. mean item, Worry v. Worry/Helplessness About Low Blood Glucoses and Worry About Social Consequences of Low Blood Glucose, number of items used] so that comparisons between studies can be made.
4. Identify clinical cut-points using innovative methodology such as clinical interviews to confirm clinically impairing FOH.
5. Conduct more longitudinal clinical trials to examine the impact of technology on FOH. Most technology studies include psychological questionnaires as a secondary outcome and sample sizes tend to be small, which likely limit statistical power to detect changes.
6. Conduct more longitudinal RCTs to test existing interventions with larger sample sizes and examine whether next generation cognitive behavioral therapies are effective to reduce FOH and mainatin long-term gains of interventions.

## Key References

- Cobry EC, Kanapka LG, Cengiz E, Carria L, Ekhlaspour L, Buckingham BA, et al. Health-Related Quality of Life and Treatment Satisfaction in Parents and Children with Type 1 Diabetes Using Closed-Loop Control. Diabetes Technol Ther. 2021;23(6):401-9.
  - This is one of the only diabetes technology studies to examine change in FOH using the Maintain High Blood Glucose, Worry/Helplessness About Low Blood Glucose, and Worry about Negative Social Consequences of a Low Blood Glucose subscales from the PHFS and CHFS.
- de Beaufort C, Schierloh U, Thankamony A, Ware J, Wilinska ME, Frohlich-Reiterer E, et al. Cambridge Hybrid Closed-Loop System in Very Young Children With Type 1 Diabetes Reduces Caregivers' Fear of Hypoglycemia and Improves Their Well-Being. Diabetes Care. 2022;45(12):3050-3.
  - This study demonstrates improved FOH in caregivers of young children who were randomized to the hybrid closed loop system compared to a sensor augmented pump group.
- Monzon AD, Cushing CC, McDonough R, Clements M, Gonder-Frederick L, Patton SR. The Development and Initial Validation of Items to Assess Parent Fear of Nighttime Hypoglycemia. J Pediatr Psychol. 2023;48(7):645-54.
  - This study established the psychometric properties of a new validated subscale – Nighttime Worry to be used with the PHFS subscales of Maintain High Blood Glucose, Worry/Helplessness About Low Blood Glucose, and Worry about Negative Social Consequences of a Low Blood Glucose.
- O'Donnell HK, Bennett Johnson S, Sileo D, Majidi S, Gonder-Frederick L, Driscoll KA. Psychometric Properties of the Hypoglycemia Fear Survey in a Clinical Sample of Adolescents with Type 1 Diabetes and Their Caregivers. J Pediatr Psychol. 2022;47(2):195–205.
  - This study provides psychometric validation of the PHFS and CHFS subscales of Maintain High Blood Glucose, Worry/Helplessness About Low Blood Glucose, and Worry about Negative Social Consequences of a Low Blood Glucose in a large clinical sample of youth with T1D and their caregivers.
- O'Donnell HK, Johnson SB, Driscoll KA. The Maintain High Blood Glucose subscale of the child hypoglycemia fear survey: proposed preliminary cut points for screening youth with type 1 diabetes. J Pediatr Psychol. 2024;499(6):421-8.
  - This study provides a clinical cut-point for the CHFS subscales Maintain High Blood Glucose.
- Patton SR, Clements MA, Marker AM, Nelson EL. Intervention to reduce hypoglycemia fear in parents of young kids using video-based telehealth (REDCHiP). Pediatr Diabetes. 2021;21(1):112-9.
  - Describes the first study to provide FOH intervention for parents of young children using cognitive behavioral therapy principles.
- Verbeeten KC, Perez Trejo ME, Tang K, Chan J, Courtney JM, Bradley BJ, et al. Fear of hypoglycemia in children with type 1 diabetes and their parents: Effect of pump therapy and continuous glucose monitoring with option of low glucose suspend in the CGM TIME trial. Pediatric diabetes. 2021;22(2):288-93.
  - This study is one of the longest longitudinal diabetes technology RCTs (12 months) that demonstrates improved FOH in a large sample of youth with T1D and their caregivers.

## Data Availability

No datasets were generated or analysed during the current study.
